# Virtual Double-System Single-Box for Absolute Dissociation Free Energy Calculations in GROMACS

**DOI:** 10.1021/acs.jcim.1c00909

**Published:** 2021-11-01

**Authors:** Marina Macchiagodena, Maurice Karrenbrock, Marco Pagliai, Piero Procacci

**Affiliations:** † Dipartimento di Chimica “Ugo Schiff”, Università degli Studi di Firenze, Via della Lastruccia 3, 50019 Sesto Fiorentino, Italy

## Abstract

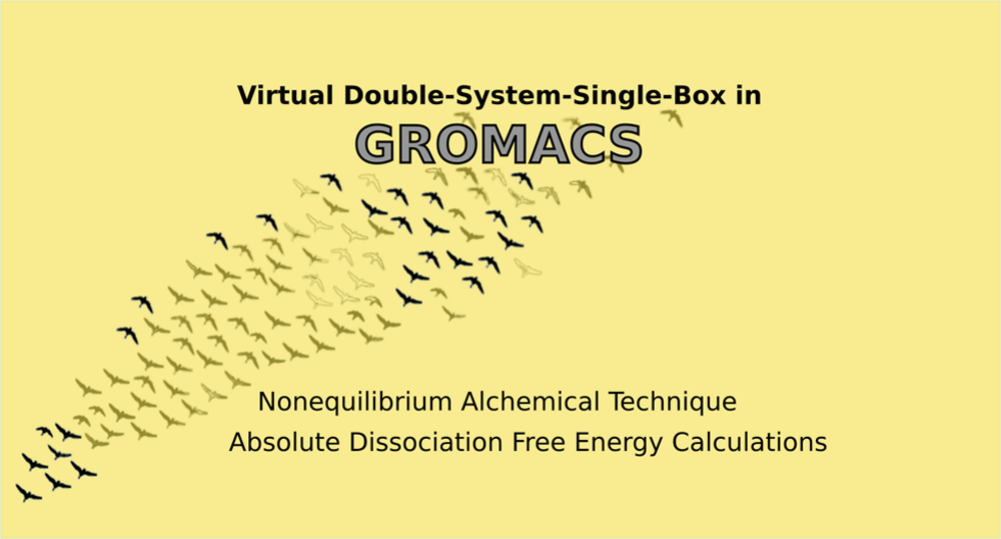

We describe a step-by-step protocol for the computation of absolute dissociation free energy with GROMACS code and PLUMED library, which exploits a combination of advanced sampling techniques and nonequilibrium alchemical methodologies. The computational protocol has been automated through an open source Python middleware (HPC_Drug) which allows one to set up the GROMACS/PLUMED input files for execution on high performing computing facilities. The proposed protocol, by exploiting its inherent parallelism and the power of the GROMACS code on graphical processing units, has the potential to afford accurate and precise estimates of the dissociation constants in drug-receptor systems described at the atomistic level. The procedure has been applied to the calculation of the absolute dissociation free energy of PF-07321332, an oral antiviral proposed by Pfizer, with the main protease (3CL^pro^) of SARS-CoV-2.

## 1 Introduction

In the last decades various computational methodologies, based on molecular dynamics (MD) simulations with explicit solvent, have been devised to improve (beyond the traditional docking approach) the calculation of absolute dissociation free energy in drug receptor systems. In this context, the so-called alchemical approach^1^ emerged recently as one of the best-automated and most widely used MD-based free energy methods. The alchemical protocol relies on a stratification of nonphysical intermediate states gradually connecting the ligand-environment potential energy function of the target end states,^2^ recovering the corresponding free energy change by a sum of free energy contributions evaluated along the stratification using free energy perturbation (FEP).^3^ Alchemical FEP, due to well known sampling issues especially at low ligand-environment coupling,^4^ is generally applied in industrial applications to the calculation of *relative* binding free energies (RBFE),^5−8^ whereby a ligand is *transmuted* into a strictly congeneric compound.

As recently noted,^8,9^ a reliable estimate of the *absolute* dissociation free energy (ADFE) via MD is a key requirement for virtual screening campaigns in industrial and academic settings in order to evaluate distant hits that are not easily amenable for RBFE calculations. Here, we present the fine-tuning of nonequilibrium (NE) alchemical transformations for ADFE calculations using the program GROMACS^10^ on high performing computing (HPC) platforms. The approach is called NEW-vDSSB. The acronym NEW^2^ stands for Non-Equilibrium Work. The work histograms, obtained by a swarm of rapid and independent NE alchemical trajectories connecting the target thermodynamic end states, are related to the corresponding free energy via the Jarzynski^11^ and Crooks^12^ theorems. vDSSB stands for *virtual* double-system single-box, a technique recently developed by some of us^13−15^ as a variant of the so-called double-system single-box (DSSB) method.^16,17^

NEW-vDSSB has been first implemented in the ORAC molecular dynamics package^18^ and has been successfully applied, for example, to the calculation of absolute binding free energy (ABFE) of ligands of the SARS-CoV-2 main protease.^13^ In this note, we describe an automated protocol, managed by a Python middleware (HPC_Drug), for implementing the NEW-vDSSB method on heterogeneous (GPU/CPU) HPC facilities using the GROMACS popular molecular dynamics package^10,19,20^ and the PLUMED library,^21,22^ requiring no intervention on the GROMACS source code.

The paper is organized as follows: we first provide some basic information on the fast switching alchemical technique employed for ADFE calculations. We then describe the computational steps to use NEW-vDSSB methodology with GROMACS. For each step, we make available the corresponding input files locally produced by the automated HPC_Drug Python middleware for execution on the remote HPC facility. In the last section, as a real-world example, we have calculated on the CINECA^23^ Marconi100 CPU-GPU heterogeneous platform the ADFE of PF-07321332 against the SARS-CoV-2 main protease (3CL^pro^). PF-07321332 is a 3CL^pro^ inhibitor proposed by Pfizer and exhibiting nanomolar affinity and capable of suppressing virus replication in human cells at submicromolar concentrations.^24^

## 2 Background

Our protocol for ADFE calculation is based on the vDSSB approach. In brief, vDSSB is a NE variant of the alchemical technique whereby the ADFE is computed in a thermodynamic cycle as the difference of the ligand solvation free energy in the bulk solvent and in the solvated complex. vDSSB relies on the *enhanced sampling* of the unbound (decoupled ligand in bulk) and bound (coupled protein-bound ligand) end states, followed by the NE step, consisting of the production of two swarms of NE-independent trajectories where the ligand is gradually recoupled (unbound leg) and decoupled (bound leg) via a driven alchemical parameter λ, producing two samples of *n*_*u*_ growth and *n*_*b*_ annihilation work values. The *n*_*b*_ and *n*_*u*_ work values are combined as two independent random variables obtaining *n*_*b*_ × *n*_*u*_ work values, referring to the process of bringing the ligand *from* the bound state *to* the bulk solvent. The convolution of the two work histograms allows us to increase significantly the resolution of the resulting work histogram with benefit for accuracy and precision of the ADFE estimate. At variance with DSSB, in vDSSB, different protocols for the bound and the unbound state can be used without violation of the Crooks^12^ and Jarzynski^11^ theorems, hence allowing us to choose the optimal box size and time protocol according to the physical dimension of the solute and the nature of the environment. As such, vDSSB is an inherently parallel algorithm, affording very efficient performances on massively parallel facilities. The free energy and the corresponding confidence interval are recovered, in a fast postprocessing stage, by constructing the convolution of the recoupling (unbound leg) and decoupling (bound leg) work histograms to which the Jarzynski theorem is applied and by correcting for a standard state-dependent term related to the translational binding site volume. For further details on vDSSB theory and technicalities, we refer to refs (13, 15, and 25). vDSSB, as any other MD-based technology for ADFE calculations, requires expertise and knowledge, which may deter the average end user. In the following, we describe a step-by-step protocol where the most complex tasks in the vDSSB workflow have been automated by an *ad hoc* devised Python middleware.

## 3 ADFE Calculation Workflow

**Figure 1:**
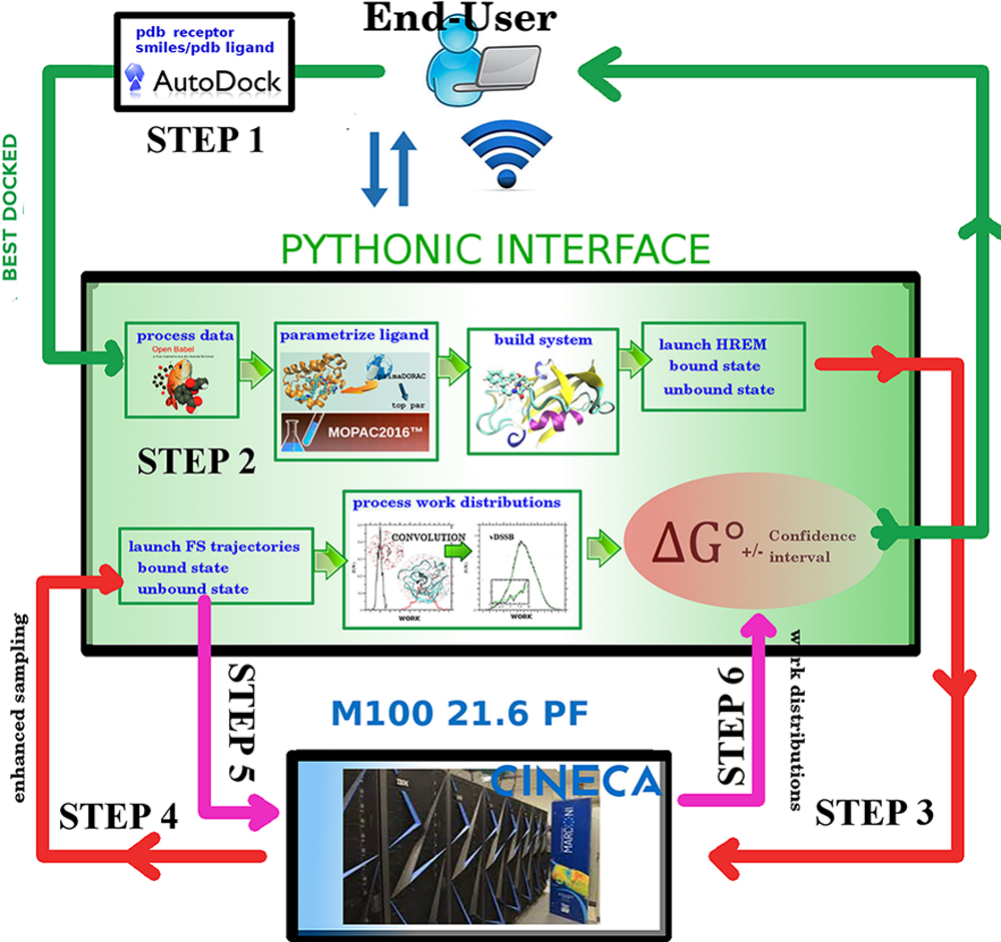
HPC_Drug/vDSSB layout for ADFE calculations: Dark green lines define end user minimal tasks. Light green arrows define tasks and data flow within the Python HPC_Drug automaton. Red (HREM) and magenta lines (FSDAM) represent connections and data flow between the Python library and the underlying HPC.

The vDSSB procedure for the HPC calculation of ADFEs (schematically described in Figure 1) is made up of six consecutive steps, starting from the 3D structure of receptor and ligand. In the following, we provide an overview of the vDSSB steps to arrive at the ADFE estimate. A companion step-by-step technical tutorial for the calculation of the ADFE in the PF-07321332–3CL^pro^ system is provided in the Supporting Information (SI). The step-by-step tutorial can be also accessed at the https://procacci.github.io/vdssb_gromacs/ site.

### 3.1 Step 1: Docking (Local)

Assumining that the experimental cocrystal structure of the complex structure is not available, we generate the initial bound state pose by way of a preliminary docking calculation of the ligand–receptor system using Autodock4.^26^ Starting from the receptor pdb code and the ligand SMILES or pdb structure, the docking step can be completed in few minutes for a typical target of 5000 atoms on a local workstation using the application script docking.bash for Unix platforms providing a convenient interface for Autodock4. The script is included in the SI with details on docking parameters. The lowest energy docked conformation is the “best-docked” bound state conformation for following vDSSB steps (used as complex with receptor, complex_ligand.pdb).

### 3.2 Step 2: HPC_Drug for HREM setup (Local)

Starting from the “best-docked” conformation generated in step 1 (complex_ligand.pdb), we produce the GROMACS input files to perform Hamiltonian Replica Exchange (HREM) MD simulation for HPC execution of the target bound and unbound states. vDSSB HREM for the bound state relies on a definition of the “hot zone” comprising the ligand and nearby residues where the enhanced sampling is implemented. The hot zone for the unbound state is the ligand itself. These complex steps, which are central in the vDSSB approach, are assisted by the HPC_Drug middleware,^27^ an effective tool for the guided submission of vDSSB job on a HPC system.

HPC_Drug is a Python application distributed under the AGPL that can be cloned from the github repository https://github.com/MauriceKarrenbrock/HPC_Drug. All installation instructions are detailed at the GitHub site. HPC_Drug reads a user-defined input file with minimal information (receptor–ligand pdb (complex_ligand.pdb) file, force field, water model, pH, etc.). For complex operations (e.g., replica exchange setup and NE input files productions), HPC_Drug has a solid default behavior, which can be changed by the end user in the main HPC_Drug input file, if necessary. Once started, HPC_Drug performs, *with no further user intervention*, a series of operations consisting of (i) the generation of the GROMACS topology files using PrimaDORAC^28^ as the default tool for the ligand force field parametrization, (ii) a preliminary minimization of the complex with the user-selected force field, (iii) the NPT equilibration in standard conditions of the resulting solvated receptor–ligand structure (using as default the TIP3P^29^ model for water or a user-selected model) in a optimal MD box, and (iv) the production of the input files (ready for use) to perform HREM simulations for bound and unbound ligand state on the HPC platforms. The GROMACS HREM input files automatically define the hot zone for the bound state based on ligand–residue distance criteria.^30^ The hot zone is implemented generating multiple topology files along the replica progression via PLUMED,^21,22^ a community-wide open source library for enhanced sampling techniques and free energy methods. PLUMED is used by HPC_Drug as a standalone tool. For HPC execution, GROMACS needs to be patched with PLUMED. Most of HPC systems for scientific applications provide modules for executing jobs with GROMACS/PLUMED.

HPC_Drug produces the mdp GROMACS main input file for HREM execution of the bound state with a series of “pulling” directives whereby the ligand is tethered to the receptor via a weak harmonic potential acting on the centers of mass (COM) of the partners. These directives generate output files, during the HREM simulation step, containing information about the COM–COM distance distributions, allowing us to calculate the binding site volume correction for the dissociation free energy.^13,15^

The final output of HPC_Drug (produced in few minutes on a local workstation for a typical system with 50,000 atoms) consists of two directories, one for the bound state and the other for the gas-phase unbound state, where all necessary data for HREM execution on a HPC (including examples of batch submission scripts) are stored. As previously stated, some advanced options (like the number of HREM replicates, number of replica, and HREM simulation time) can be changed in the input file. An example of HPC_Drug input file is reported in the SI.

### 3.3 Step 3: HREM Simulations (HPC)

Upon uploading on the remote HPC platform the two HPC_Drug generated HREM directories, the HREM simulations can be straightforwardly executed on the HPC system. In the SI, we included the batch script used to run the bound state HREM simulation on the heterogeneous Marconi100 HPC platform (CINECA), equipped with 4 Nvidia VOLTA GPU per node. In particular we ran six batteries (replicates) each of 24 replica on 36 nodes (144 Nvidia VOLTA GPU). For the unbound state, we perform the HREM for a ligand molecule in gas phase; we ran four batteries each of eight replicas on eight nodes (32 Nvidia VOLTA GPU).

### 3.4 Step 4: Selection of (Enhanced Sampled) Configurations

This postprocessing operation is conveniently done on the HPC system. A preliminary analysis of the “pulling” output files in target state replicas might be needed to verify that the ligand did not leave the binding site despite the weak ligand–receptor tethering restraint. No less than 200 configurations files are extracted using the GROMACS tools from the target state HREM trajectories xtc files for the bound and unbound states. Simple bash scripts to harvest configurations at regular intervals are provided in the SI. For the unbound state, the gas-phase HREM configurations of the ligand (gro files) are combined with a well-equilibrated box of water molecules.

### 3.5 Step 5: Fast Switching Alchemical Simulations (HPC)

#### 3.5.1 Bound State

The ligand decoupling in the bound state, starting from the HREM-generated pdb configurations, is straightforwardly implemented via the multidir GROMACS option whereby the independent alchemical trajectories are originated from the HREM-generated pdb configurations in dedicated directories. In the HPC submission script for Marconi100 (see the SI), each *independent* trajectory on the MPI layer is assigned to a GPU, in a very effective hybrid parallelization scheme. The resulting embarrassingly parallel run engages as many GPUs (MPI processes) as HREM-generated pdb files. According to a consensus protocol,^2^ the alchemical decoupling, for each independent trajectory, is done in two consecutive annihilation steps and hence using two GROMACS mdp files (provided in the SI). In the first and second steps, we switch off the electrostatic ligand-environment interactions and the Lennard-Jones (LJ) potential, respectively, using a soft-core regularization.^31^ Separate estimates of the electrostatic and LJ contributions to the dissociation free energy are useful to gain hints on how to modify the molecule to increase the affinity. Typical annihilation times depends on the ligand size. For a typical drug size ligand (300:400 MW), we use 0.375 ns for the switching off the atomic charges and 0.750 ns for turning off the LJ ligand-environment interactions. Intramolecular interactions are always on during the alchemical process. An example of the batch script for submitting the fast-switching parallel job for the bound state is provided in the SI.

#### 3.5.2 Unbound State

The fast switching growth run is technically similar to the bound state run. In each fast-growth trajectory (MPI process), we switch on the LJ and electrostatic potential in sequence in a total time (for a typical ligand) of 0.520 ns. Further details can be found in the SI.

### 3.6 Step 6: Calculation of Dissociation Free Energy

The main output in the preceding step 5 are the dhdl.xvg files, stored in the directories specified in the multidir option. In these files, the instantaneous values of the derivative of the Hamiltonian with respect to λ at each time step are recorded. For each alchemical trajectory, the work can be computed by numerical integration. This operation is done for the two contributions, electrostatic and LJ, and for the bound and unbound transformations. The resulting *n*_*b*_ and *n_u_* total work values, referring to the bound and unbound work histograms, *P*_*b*_(*W*), *P*_*u*_(*W*), can be combined yielding *n*_*b*_ × *n*_*u*_ work data as  in the convolution (*P*_*b*_ × *P*_*u*_)(*W*) = *∫dw P*_*b*_(*W*)*P*_*u*_(*W* – *w*), affording the Jarzynski estimates of the free energy cost of transferring the ligand from the bound state to the bulk (dissociation free energy)
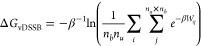
1

The confidence interval is evaluated by bootstrapping with resampling the bound and unbound work data *before* the combination of work values.

The free energy estimate can also be computed assuming that the convolution (*P*_*b*_ × *P*_*u*_)(*W*) can be represented as a Gaussian mixture using
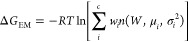
2where *c* is the number of components in the mixture,  is the normal distribution of the *i*th component with mean and variance μ_*i*_,  and *w*_*i*_ are the *i*th weights such that . The parameters in eq 2 are determined from the *n*_*b*_ × *n*_*u*_ work data using the Expectation Maximization (EM) algorithm.^32,33^Equation 2 is a direct consequence of the Crooks theorem, providing an *unbiased* alternative estimate of the dissociation free energy if the distribution is the result of a combination of normal distributions. While Δ*G*_*EM*_ does not suffer of the inherent^34^ bias of the Jarzynski estimate, eq 2 can be less precise as the EM fitting process can be ill-defined and unstable if the mixture is made by overlapping components with disparate variances. A discussion about Jarzynski and EM estimates and their differences can be found in refs (13 and 15).

All these postprocessing calculations can be performed directly on the HPC using a simple script file (works.bash) detailed in the SI.

Finally, the standard dissociation free energy can be calculated by summing to Δ*G*_vDSSB_ the volume correction and the finite-size correction, when a ligand is not electrically neutral.^35,36^ The volume correction, evaluated from the variance of COM–COM distribution, is calculated as
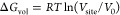
3with *V*_0_ = 1661 Å and , where σ^2^ is the variance of the COM–COM distribution in the HREM stage obtained starting from pullx.xvg files. The finite-size correction, necessary only in case of charged ligands, is calculated applying the following equation
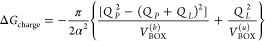
4where *Q*_*P*_ and *Q*_*L*_ are the net charge on the receptor and ligand, respectively,  are the MD box volumes of the bound and unbound states, and α is the Ewald convergence parameter. Summarizing, the vDSSB estimate of the standard dissociation energy is given by
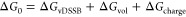
5

For further technical details on the postprocessing stage for ADFE calculations, see the SI.

## 4 Application

**Figure 2:**
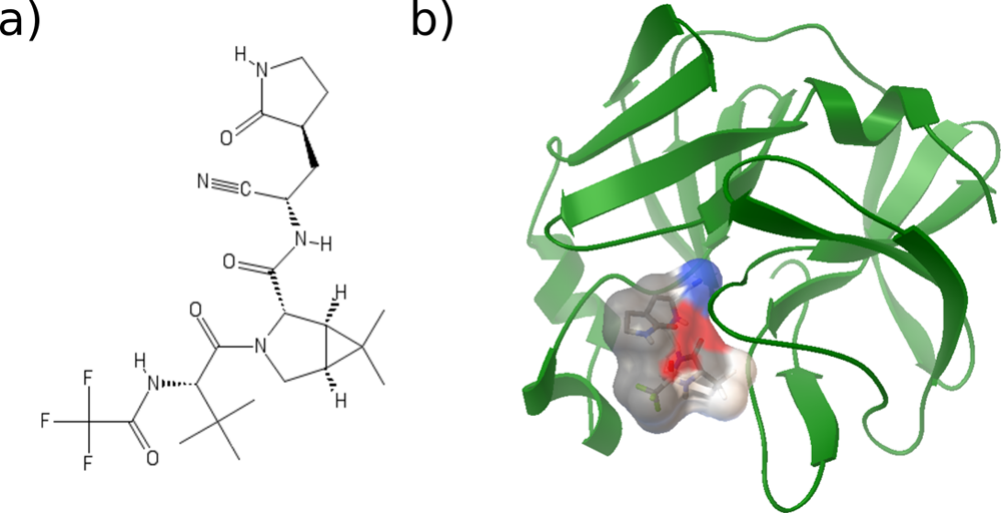
(a) Structure of PF-07321332. (b) Ligand–receptor complex with lowest docking binding free energy, “best-docked”. The catalytic dyad (HIS41-CYS145) has been considered in the neutral state.^13^ The surface shows the interactions between PF-07321332 and 3CL^pro^.

During the Covid-19 pandemic, considerable efforts have been devoted to the identification of an effective antiviral agent for SARS-CoV-2 via computational approaches.^37^ Here, we report on the calculation of the dissociation affinity of the PF-07321332 compound (structure in Figure 2a) against the main protease (3CL^pro^) of SARS-CoV-2 using the vDSSB technique.

Starting from the protein X-ray structure^38^ and the PubChem^39^ generated SMILES code of PF-07321332, we performed the docking calculation on the catalytic domains I + II of 3CL^pro^ obtaining as the best docking pose with a binding energy value of −9.07 kcal/mol the structure of the ligand–receptor complex reported in Figure 2b (step 1).

Using this starting configuration, the Python application generates the two directories to perform HREM of bound and unbound states (step 2).

The two HPC_Drug-generated directories are then transferred to the HPC system (CINECA Marconi100). On the HPC, the bound HREM was carried out using six batteries and 24 replicas on 144 GPUs collecting about 142 ns on the target state in 24 h, while for the unbound HREM we launched four batteries and eight replicas on 32 GPUs, collecting 32 ns in about 2 h (step 3).

**Figure 3:**
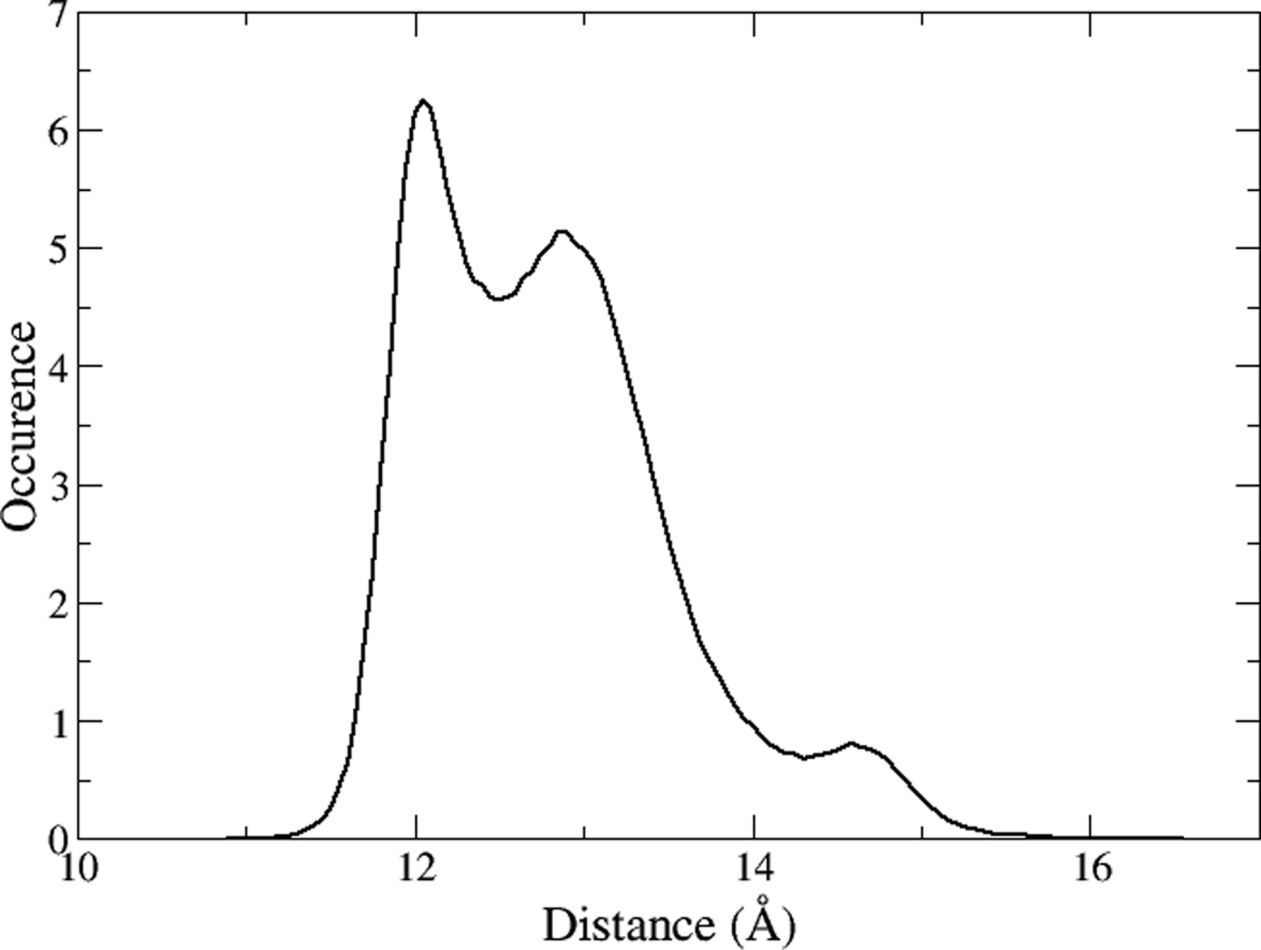
COM–COM distribution obtained from bound state HREM trajectories.

From the “pulling” directive output in the target states of the bound HREM directory, we computed the COM receptor–ligand distribution (Figure 3) to obtain the volume correction to the dissociation free energy. From the GROMACS xtc files in the bound HREM, we extracted 384 configurations of the target state sampled at regular intervals as starting points for the subsequent NE simulations corresponding to the fast annihilation of the ligand in the bound state. From the gas-phase HREM target state trajectories, we extracted 192 configurations of the ligand. These conformations were combined with a well-equilibrated water box to provide the starting structures for the ligand growth NE process in the solvent (step 4).

The fast switching stage produced 384 NE bound state trajectories lasting ≃1.2 ns and engaging as many GPUs in about 2 h and 192 NE 0.520 ns unbound state trajectories on a many GPUs in about 20 min (step 5).

The dhdl files produced in step 5 were finally postprocessed on the HPC using the work.bash script obtaining the fast switching work values for bound (annihilation) and unbound (growth) states along with the estimates of the dissociation free energy Δ*G*_vDSSB_ and Δ*G*_EM_ in eqs1 and 2, respectively (step 6).

**Figure 4:**
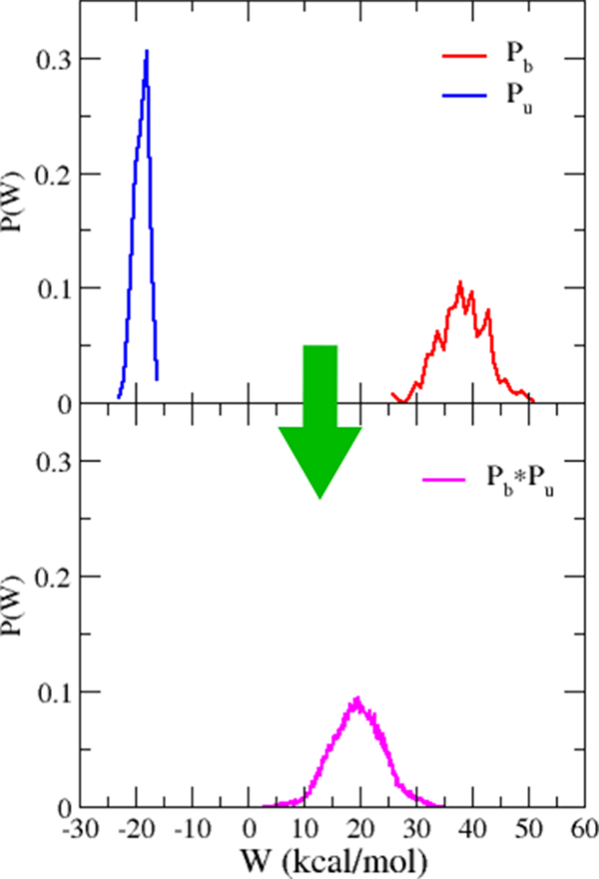
Upper panel: *P*_*u*_(*W*), *P*_*b*_(*W*) work histograms obtained from NE decoupling (bound) and recoupling (unbound) runs. Lower panel: (*P*_*b*_ × *P*_*u*_)(*W*) convolution process.

In Figure 4, we report the work distributions for bound and unbound state obtained and the convolution calculated by combining the bound and unbound work samples. The correction volume, obtained applying eq 3 and analyzing the Figure 3, is of −2.81 kcal/mol. The finite-charge correction is null since PF-07321332 is a neutral molecule. The final dissociation free energy estimate (Δ*G*_vDSSB_, eq 1) is 5.8 ± 0.9 kcal/mol.

## 5 Conclusions

We have described a step-by-step protocol for the calculations of the absolute dissociation free energy calculation using the GROMACS code and the PLUMED library, relying on the end states enhanced sampling with HREM followed by the production of concurrent non equilibrium fast switching alchemical trajectories. The approach emulates a double-system single-box ligand–receptor dissociation process by effectively combining the ligand annihilation work in the bound state with the ligand growth work in the bulk solvent. The methodology, specifically tailored for massively parallel architectures, allows us to obtain a reliable estimate of the ligand–receptor ADFE on GPU-accelerated HPC architectures in about 24 h of wall clock. HPC submission of the complex HREM computational stage with GROMACS is greatly facilitated by the Python middleware HPC_Drug, freely available at the GitHub site. A detailed tutorial is provided in the Supporting Information where the noncovalent dissociation affinity of the PF-07321332 inhibitor of the SARS-CoV-2 main protease is evaluated.

## 6 Data and Software Availability

HPC_Drug is publicly available at https://github.com/MauriceKarrenbrock/HPC_Drug under a AGPL license. The simulations have been performed using GROMACS (https://www.gromacs.org/) and the PLUMED library (https://www.plumed.org/). Autodock4 (http://autodock.scripps.edu/) has been adopted for preliminary docking calculations. ORAC software (http://www1.chim.unifi.it/orac/) has been used for ligand force field parameters. All homemade scripts used to calculate PF-07321332–3CL^pro^ absolute dissociation free energy are provided in the zip file in the Supporting Information and described in the pdf file in the Supporting Information. The step-by-step tutorial can be accessed at the https://procacci.github.io/vdssb_gromacs/ site. All output files are reported on https://zenodo.org/record/5139374.

## Supporting Information Available

Step-by-step technical tutorial for the calculation of the ADFE in the PF-07321332–3CL^pro^ system (PDF)
All homemade scripts used to calculate PF-07321332–3CL^pro^ absolute dissociation free energy (ZIP)

## References

[ref1] Gao J.; Kuczera K.; Tidor B.; Karplus M. Hidden Thermodynamics of Mutant Proteins: a Molecular Dynamics Analysis. Science 1989, 244, 1069–1072. 10.1126/science.2727695. 2727695

[ref2] Pohorille A.; Jarzynski C.; Chipot C. Good Practices in Free-Energy Calculations. J. Phys. Chem. B 2010, 114, 10235–10253. 10.1021/jp102971x. 20701361

[ref3] Zwanzig R. W. High-temperature Equation of State by a Perturbation Method. I. Nonpolar Gases. J. Chem. Phys. 1954, 22, 1420–1426. 10.1063/1.1740409.

[ref4] Pal R. K.; Gallicchio E. Perturbation Potentials to Overcome Order/Disorder Transitions in Alchemical Binding Free Energy Calculations. J. Chem. Phys. 2019, 151, 124116 10.1063/1.5123154. 31575187

[ref5] Wang L.; Wu Y.; Deng Y.; Kim B.; Pierce L.; Krilov G.; Lupyan D.; Robinson S.; Dahlgren M. K.; Greenwood J.; Romero D. L.; Masse C.; Knight J. L.; Steinbrecher T.; Beuming T.; Damm W.; Harder E.; Sherman W.; Brewer M.; Wester R.; Murcko M.; Frye L.; Farid R.; Lin T.; Mobley D. L.; Jorgensen W. L.; Berne B. J.; Friesner R. A.; Abel R. Accurate and Reliable Prediction of Relative Ligand Binding Potency in Prospective Drug Discovery by Way of a Modern Free-Energy Calculation Protocol and Force Field. J. Am. Chem. Soc. 2015, 137, 2695–2703. 10.1021/ja512751q. 25625324

[ref6] Cui G.; Graves A. P.; Manas E. S. GRAM: A True Null Model for Relative Binding Affinity Predictions. J. Chem. Inf. Model. 2020, 60, 11–16. 10.1021/acs.jcim.9b00939. 31874032

[ref7] Kuhn M.; Firth-Clark S.; Tosco P.; Mey A. S. J. S.; Mackey M.; Michel J. Assessment of Binding Affinity via Alchemical Free-Energy Calculations. J. Chem. Inf. Model. 2020, 60, 3120–3130. 10.1021/acs.jcim.0c00165. 32437145

[ref8] Cournia Z.; Allen B. K.; Beuming T.; Pearlman D. A.; Radak B. K.; Sherman W. Rigorous Free Energy Simulations in Virtual Screening. J. Chem. Inf. Model. 2020, 60, 4153–4169. 10.1021/acs.jcim.0c00116. 32539386

[ref9] Procacci P. Methodological Uncertainties in Drug-Receptor Binding Free Energy Predictions Based on Classical Molecular Dynamics. Curr. Opin. Struct. Biol. 2021, 67, 127–134. 10.1016/j.sbi.2020.08.001. 33220532

[ref10] Páll S.; Zhmurov A.; Bauer P.; Abraham M.; Lundborg M.; Gray A.; Hess B.; Lindahl E. Heterogeneous Parallelization and Acceleration of Molecular Dynamics Simulations in GROMACS. J. Chem. Phys. 2020, 153, 134110 10.1063/5.0018516. 33032406

[ref11] Jarzynski C. Nonequilibrium Equality for Free Energy Differences. Phys. Rev. Lett. 1997, 78, 2690–2693. 10.1103/PhysRevLett.78.2690.

[ref12] Crooks G. E. Nonequilibrium Measurements of Free Energy Differences for Microscopically Reversible Markovian Systems. J. Stat. Phys. 1998, 90, 1481–1487. 10.1023/A:1023208217925.

[ref13] Macchiagodena M.; Pagliai M.; Karrenbrock M.; Guarnieri G.; Iannone F.; Procacci P. Virtual Double-System Single-Box: A Nonequilibrium Alchemical Technique for Absolute Binding Free Energy Calculations: Application to Ligands of the SARS-CoV-2 Main Protease. J. Chem. Theory Comput. 2020, 16, 7160–7172. 10.1021/acs.jctc.0c00634. 33090785 PMC8015232

[ref14] Procacci P. A. Remark on the Efficiency of the Double-System/Single-Box Nonequilibrium Approach in the SAMPL6 SAMPLing Challenge. J. Comput.-Aided Mol. Des. 2020, 34, 635–639. 10.1007/s10822-020-00312-2. 32277315

[ref15] Macchiagodena M.; Karrenbrock M.; Pagliai M.; Guarnieri G.; Iannone F.; Procacci P. Nonequilibrium Alchemical Simulations for the Development of Drugs Against Covid-19; Springer US: New York, pp 1–41.

[ref16] Gapsys V.; Michielssens S.; Peters J.; de Groot B.; Leonov H. Calculation of Binding Free Energies. In Molecular Modeling of Protein; Humana Press, 2015; pp 173–209. 10.1007/978-1-4939-1465-4_9 25330964

[ref17] Rizzi A.; Jensen T.; Slochower D. R.; Aldeghi M.; Gapsys V.; Ntekoumes D.; Bosisio S.; Papadourakis M.; Henriksen N. M.; de Groot B. L.; Cournia Z.; Dickson A.; Michel J.; Gilson M. K.; Shirts M. R.; Mobley D. L.; Chodera J. D. The SAMPL6 SAMPLing Challenge: Assessing the Reliability and Efficiency of Binding Free Energy Calculations. J. Comput.-Aided Mol. Des. 2020, 34, 601–633. 10.1007/s10822-020-00290-5. 31984465 PMC7282318

[ref18] Procacci P. Hybrid MPI/OpenMP Implementation of the ORAC Molecular Dynamics Program for Generalized Ensemble and Fast Switching Alchemical Simulations. J. Chem. Inf. Model. 2016, 56, 1117–1121. 10.1021/acs.jcim.6b00151. 27231982

[ref19] Hess B.; Kutzner C.; van der Spoel D.; Lindahl E. GROMACS 4: Algorithms for Highly Efficient, Load-Balanced, and Scalable Molecular Simulation. J. Chem. Theory Comput. 2008, 4, 435–447. 10.1021/ct700301q. 26620784

[ref20] Abraham M. J.; Murtola T.; Schulz R.; Páll S.; Smith J. C.; Hess B.; Lindahl E. GROMACS: High Performance Molecular Simulations Through Multi-Level Parallelism from Laptops to Supercomputers. SoftwareX 2015, 1–2, 19–25. 10.1016/j.softx.2015.06.001.

[ref21] Tribello G. A.; Bonomi M.; Branduardi D.; Camilloni C.; Bussi G. PLUMED 2: New Feathers for an Old Bird. Comput. Phys. Commun. 2014, 185, 604–613. 10.1016/j.cpc.2013.09.018.

[ref22] Bussi G. PLUMED.PDB, 2020. https://github.com/plumed/tuto-trieste-instructions (accessed 8 September 2021).

[ref23] Erbacci G. Trends in HPC Architectures and Parallel Programmming; PRACE Winter School, 2012. https://materials.prace-ri.eu/137/1/ParallelArchitectures-Erbacci.pdf (accessed 22 January 2016).

[ref24] Halford B. Pfizer Unveils its Oral SARS-CoV-2 Inhibitor. Chem. Eng. News 2021, 7 10.47287/cen-09913-scicon3.

[ref25] Procacci P.; Macchiagodena M.; Pagliai M.; Guarnieri G.; Iannone F. Interaction of Hydroxychloroquine with SARS-CoV2 Functional Proteins Using All-Atoms Non-Equilibrium Alchemical Simulations. Chem. Commun. 2020, 56, 8854–8856. 10.1039/D0CC03558K. 32633733

[ref26] Trott O.; Olson A. J. AutoDock Vina: Improving the Speed and Accuracy of Docking with a New Scoring Function, Efficient Optimization, and Multithreading. J. Comput. Chem. 2010, 31, 455–461. 10.1002/jcc.21334. 19499576 PMC3041641

[ref27] Karrenbrock M. HPC_Drug: a Python Application for Drug Development on High Performance Computing Platforms. M.Sc. Thesis, Università degli Studi di Firenze, Firenze, Italy, 2020.

[ref28] Procacci P. PrimaDORAC: A Free Web Interface for the Assignment of Partial Charges, Chemical Topology, and Bonded Parameters in Organic or Drug Molecules. J. Chem. Inf. Model. 2017, 57, 1240–1245. 10.1021/acs.jcim.7b00145. 28586207

[ref29] Jorgensen W. L.; Chandrasekhar J.; Madura J. D.; Impey R. W.; Klein M. L. Comparison of Simple Potential Functions for Simulating Liquid Water. J. Chem. Phys. 1983, 79, 926–935. 10.1063/1.445869.

[ref30] Wang L.; Friesner R. A.; Berne B. J. Replica Exchange with Solute Scaling: A More Efficient Version of Replica Exchange with Solute Tempering (REST2). J. Phys. Chem. B 2011, 115, 9431–9438. 10.1021/jp204407d. 21714551 PMC3172817

[ref31] Beutler T.; Mark A.; van Schaik R.; Gerber P.; van Gunsteren W. Avoiding Singularities and Numerical Instabilities in Free Energy Calculations Based on Molecular Simulations. Chem. Phys. Lett. 1994, 222, 529–539. 10.1016/0009-2614(94)00397-1.

[ref32] Dempster A.; Laird N.; Rubin D. Maximum Likelihood from Incomplete Data via the EM Algorithm. J. Royal Stat. Soc. B 1977, 39, 1–38. 10.1111/j.2517-6161.1977.tb01600.x.

[ref33] Gupta M. R.; Chen Y. Theory and Use of the EM Algorithm. Found. Trends Signal Process. 2010, 4, 223–296. 10.1561/2000000034.

[ref34] Gore J.; Ritort F.; Bustamante C. Bias and Error in Estimates of Equilibrium Free-Energy Differences from Nonequilibrium Measurements. Proc. Natl. Acad. Sci. U. S. A. 2003, 100, 12564–12569. 10.1073/pnas.1635159100. 14528008 PMC240657

[ref35] Darden T.; Pearlman D.; Pedersen L. G. Ionic Charging Free Energies: Spherical Versus Periodic Boundary Conditions. J. Chem. Phys. 1998, 109, 10921–10935. 10.1063/1.477788.

[ref36] Procacci P.; Guarrasi M.; Guarnieri G. SAMPL6 Host–Guest Blind Predictions Using a Non Equilibrium Alchemical Approach. J. Comput.-Aided Mol. Des. 2018, 32, 965–982. 10.1007/s10822-018-0151-9. 30128927

[ref37] Chodera J.; Lee A. A.; London N.; von Delft F. Crowdsourcing Drug Discovery for Pandemics. Nat. Chem. 2020,.12 581 10.1038/s41557-020-0496-2 32555379

[ref38] Liu X.; Zhang B.; Jin Z.; Yang H.; Rao Z. 6LU7: The Crystal Structure of 2019-nCoV Main Protease in Complex with an Inhibitor N3; RSCB PDB, 2020.

[ref39] Kim S.; Thiessen P. A.; Bolton E. E.; Chen J.; Fu G.; Gindulyte A.; Han L.; He J.; He S.; Shoemaker B. A.; Wang J.; Yu B.; Zhang J.; Bryant S. H. PubChem Substance and Compound Databases. Nucleic Acids Res. 2016, 44, D1202–D1213. 10.1093/nar/gkv951. 26400175 PMC4702940

