# Supplementary material for: Virtual Double-System Single-Box for Absolute Dissociation Free Energy Calculations in GROMACS
Source: J Chem Inf Model. 2021 Nov 1;61(11):5320–6. doi: 10.1021/acs.jcim.1c00909 (PMC8611716; doi:10.1021/acs.jcim.1c00909)
Supplement: Supplementary file 1 — ci1c00909_si_001.pdf [file ci1c00909_si_001.pdf]

Supporting Information for "Virtual Double-System Single-Box for Absolute Dissociation Free Energy Calculations in GROMACS"

Marina Macchiagodena,*,† Maurice Karrenbrock,†,‡ Marco Pagliai,† and Piero Procacci*,†

†Dipartimento di Chimica "Ugo Schiff", Università degli Studi di Firenze, Via della Lastruccia 3, 50019 Sesto Fiorentino, Italy

‡Present address: Institut des Sciences Pharmaceutiques De Suisse Occidentale-Université de Genève, CMU – Rue Michel Servet 1, CH-1211 Genève 4, Switzerland

E-mail: marina.macchiagodena@unifi.it; piero.procacci@unifi.it

This Supporting Information document is conceived as a tutorial for the calculation of absolute dissociation free energy between PF-07321332 and 3CL^{pro}. The methodology is divided into six consecutive steps. For each step, we report the computational details. The major massively parallel computational step consists in the HREM simulations (Step 3) and in fast switching alchemical simulations (Step 5). The Steps 1, 2 and 4 are fast preparatory automatized procedures for running the two major computational jobs on the HPC. The last Step 6 is the automatized post-processing of the vDSSB data. All files necessary (filenames are indicated in **blue bold**) are in the compressed SI.zip archive. In the archive are present also the output file/folder as a reference to verify the success of

the protocol application (filenames in the text are in **red bold** with OUT keyword). Full output data for the application PF-07321332-3CL^{pro} can be found on the Zenodo repository (https://zenodo.org/record/5139374). The step-by-step tutorial can be also accessed at the https://procacci.github.io/vdssb_gromacs/ site.

Step 1: Docking (local)

Docking study between ligand (PF-07321332) **ligand.pdb** and receptor (3CL^{pro}, domain I+II), **3clc.pdb** is performed using Autodock4[1] with the script **docking.bash**.

docking.bash -l ligand -r 3clc -x -10.55 -y 20.65 -z 66.75 -p 60

The calculation runs 50 docking experiments using the Lamarckian genetic algorithm with the center of mass of the fully flexible ligand placed within a 22.5 Å (60 grid points, **-p 60**, spaced 0.375 Å) side-length cubic box centered at the receptor active site (**-x -10.55 -y 20.65 -z 66.75**). Up to 2.5 million energy evaluations are performed for each experiment. The results are clustered using a tolerance of 2.0 Å. The lowest energy docked conformation of the lowest energy cluster is the "best-docked" conformation. The complex structure of ligand ("best-docked") and protein, **complex_ligand.pdb_OUT**, is the starting structure for HPC_Drug.

For help about docking script, just issue the previous command without arguments (**docking.bash**).

Step 2: HPC_drug for HREM set-up (local)

All installation instructions are detailed in github repository https://github.com/MauriceKarrenbrock/HPC
and reported at the end of this file. After installation, to use HPC_Drug is necessary to activate the environment with:

conda activate HPC_Drug

Then to run HPC_Drug just type the following command using the input file **input_correct_gromacs.txt**

python /HPCDrug_INSTALL_DIRECTORY/main.py input_correct_gromacs.txt

where HPCDrug_INSTALL_DIRECTORY is the PATH where HPC_Drug is installed. The input_correct_gromacs.txt contains information about pdb file name (in our case **complex_ligand.pdb**), path, HREM options, box dimensions, water model, and so on.

HPC_Drug takes some time, at the end in the work directory there will be intermediate GROMACS files due to optimization and equilibration runs, force field files of ligand and receptor and two folders. The two folders, one for the bound and one for the unbound state, contain the files to run HREM on the HPC platform. In SI.zip we provide the two reference folders obtained for the case study PF-07321332 in complex with $3CL^{pro}$ (**3clp_HREM_OUT** and **LIG_only_ligand_HREM_OUT**).

Step 3: HREM simulations (HPC)

In the two folders generated by HPC_Drug in previous step (**3clp_HREM** and **LIG_only_ligand_HREM**) there is a script file **MAKE_TPR_FILES.sh**. This script is useful to generate all tpr files that are necessary to run the HREM simulations.

HPC_Drug generates two tentative batch files for HPC submission in the **3clp_HREM** and **LIG_only_ligand_HREM** directories, based on the syntax of the SLURM workload manager. These files must be hacked and adapted to the specific HPC platform job scheduling system by the end-user. In the reference sub-directories **bound_OUT** and **unbound_OUT** of the 3_hrem directory in the SI.zip archive, there are working SLURM submission files to execute the HREM simulation for the bound and unbound relative to the complex PF-07321332-$3CL^{pro}$ on the heterogeneous Marconi100 HPC platform (CINECA), equipped with 4 Nvidia VOLTA GPU per node. The job **HREM_input_bound.slr** and **HREM_input_unbound.slr** requests 36 nodes (144 Nvidia VOLTA GPU) and 8 nodes (32 Nvidia VOLTA

GPU), respectively. The job relative to the bound state produces about 142 ns on the target state in 24 wall clock hours, running six replicates of 24-replica exchange simulation involving a "hot-zone" including the ligand and nearby residues.

Step 4: Selection of the (enhanced sampled) configurations (HPC)

To extract the configurations from the target state of HREM we use common tools of the GROMACS suite (trjconv) automatized in scripts specified for bound and unbound state. These script are provided in the SI.zip archive and must be launched from the HPC_Drug-generated directories for the bound and unbound state after completion of the the HREM simulations. Extraction of the HREM-generated configurations for the bound and unbound state requires few seconds.

Bound state

Using the script file **getconf.sh** we extract configurations from the HREM-generated target states to perform fast switching alchemical simulations.

Unbound state

Using the script file **getconf.sh** we extract configurations from target states to perform fast switching alchemical simulations. Since the HREM simulation for unbound state is performed on a single ligand molecule in gas phase using a big box, the coordinates are recentered. Each gas phase configuration is combined with a well equilibrated box of water molecules (only_water_tip3p.gro) using the script **addwater.sh**. The file only_water_tip3p.gro is provided by HPC_Drug in Step 2.

Step 5: Fast Switching Alchemical Simulations

Once the starting phase-points have been generated in Step 4, we are ready to launch the second major computational and massively parallel stage in the vDSSB approach, namely the swarm of the bound state annihilation trajectories and the swarm of the unbound state growth trajectories.

Bound state

The annihilation trajectory are generated, in a sub-directory ("fsdam", to be created by the end-user) of the bound state directory containing the HREM data, by the application script **maketprQ.sh** provided in the SI.zip archive. In the specific example of the the complex PF-07321332-3CL^{pro}, the script generates 384 folders (**fsdam/b**i) containing the GROMACS tpr file to switch off the ligand atomic charges in 0.375 ns, starting from the configurations generated in Step 4. The fast switching simulations are submitted on the HPC using the batch submission script **submit.slr** (also provided in the SI.zip) which allows: (a) to switch off the atomic charges in 384 parallel FS trajectories according to the tpr files generated with **maketprQ.sh**; (b) to generate the new tpr files in each of **fsdam/b**i folders using as starting point the confout.gro configuration file of previous run; (c) to turn off the LJ ligand-environment interactions in 0.750 ns. **maketprQ.sh** and **submit.slr** use mdp files **transitionQ.mdp** and **transitionvdw.mdp** provided in SI.zip. The bound state ligand annihilation job required about two hours on the Marconi100 HPC platform.

Unbound state

Using the bash script **maketprvdw.sh** (provided in the SI.zip), tpr GROMACS files to perform unbound fast switching alchemical simulations are generated in the "fsdam" user-created subdirectory. The script creates 192 folders (**fsdam/u**i) containing the tpr files to switch on LJ ligand-water interactions in 0.360 ns, starting from the configurations generated in Step 4. The simulations are submitted using **submit.slr** which allows: (a) to switch on

the LJ ligand-water interactions using the tpr files generated with **maketprvdw.sh**; (b) to generate the new tpr files using as starting point the confout.gro configuration file of previous run; (c) to turn on ligand atomic charges in 0.160 ns. **maketprvdw.sh** and **submit.slr** use the mdp files **transitionvdw.mdp** and **transitionQ.mdp** provided in SI.zip. The unbound ligand growth required few wall clock minutes on the Marconi100 HPC cluster.

Step 6: Calculation of dissociation free energy

In each of the 384 bound folders (**bound/fsdam/b**i, with $1 < i < 384$) and in each of the 192 the unbound folders (**unbound/fsdam/u**i, with $1 < i < 192$) two files dhdl.xvg (**dhdlQ.xvg** and **dhdlvdw.xvg**, the first relative to electrostatic ligand-environment interactions, and the second one to LJ potential) are generated in Step 5. These files are combined to obtain the absolute dissociation free energy using the script **works.bash** which returns 4 estimates: 3 Gaussian mixtures (one, two and three components) and the Jarzynski estimate.

To calculate the volume correction, the script **VOLcor.bash** is used. It acts on **pullx.xvg** files generated in target state replica of each BATTERY. In SI.zip is also provided the script **Qcor.sh** for finite-size correction applying to charged ligand. For technical details on the usage of this scripts see the README files in the 6_post directory of the SI.zip archive.

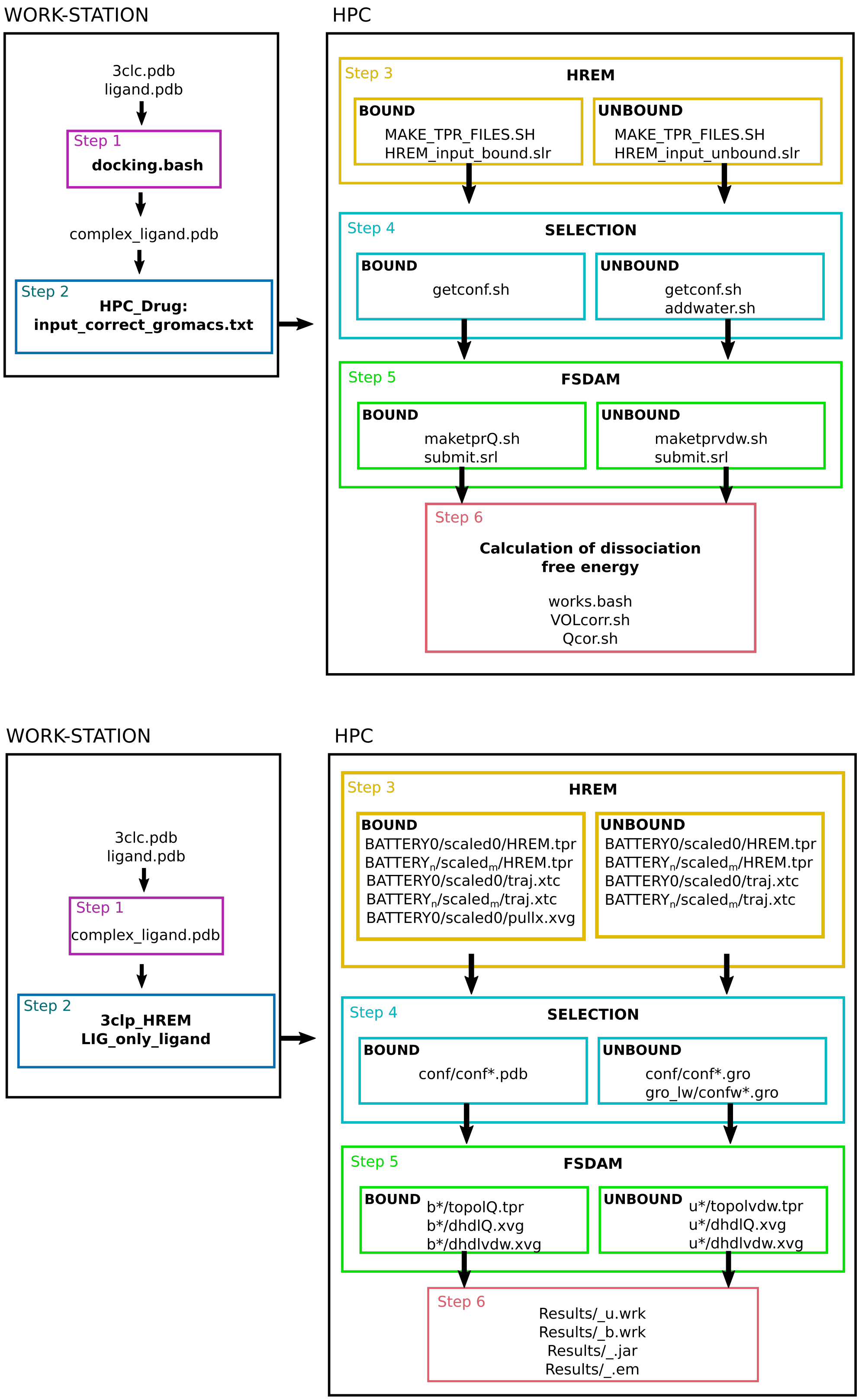

Figure S1: Graphical description of procedure. Upper panel: script and commands for each steps. Lower panel: file and results obtained in each steps.

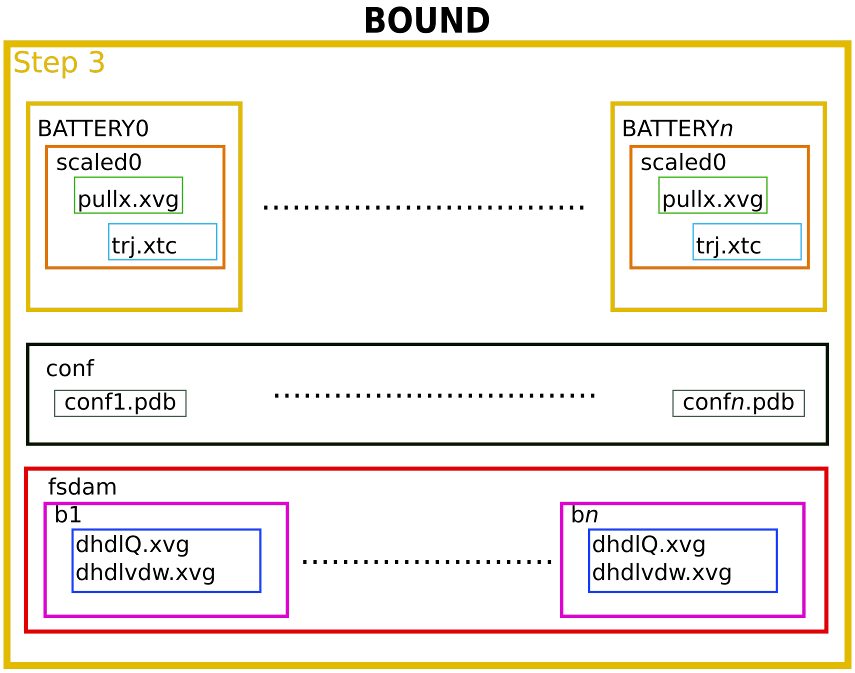

Figure S2: Graphical representation of the bound folder content.

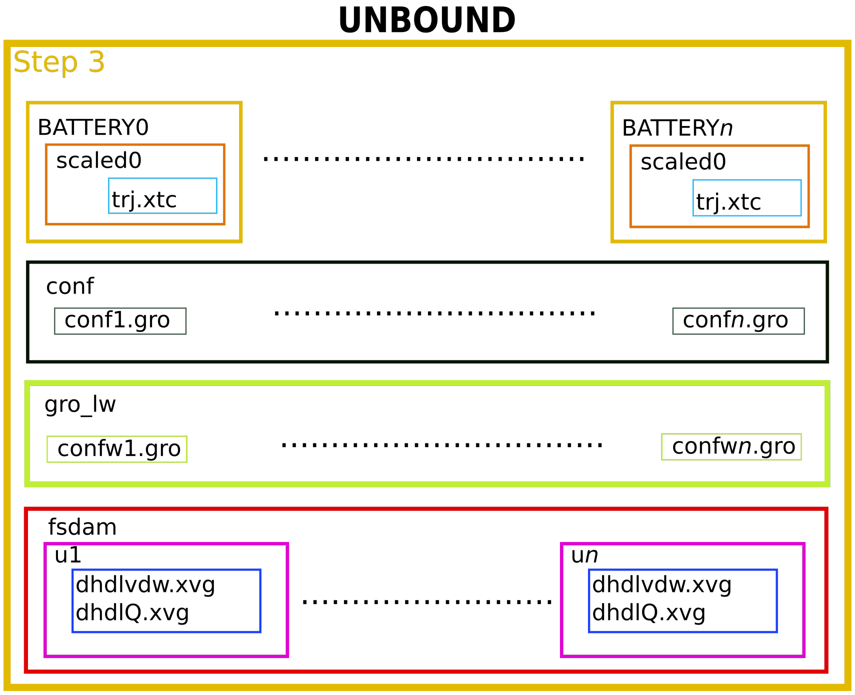

Figure S3: Graphical representation of the unbound folder content.

Install and Setup of HPC_Drug

To install the program you simply have to download it from the GitHub repository: https://github.com/MauriceKarrenbrock/HPC_Drug and, if you already had a setup environment, you could already run the main.py program (or if you would like to use one of the other scripts in the scripts/ directory remember to copy it in the root one first).

The environment setup is a little bit longer, in fact HPC_Drug, being a middleware, has a fair amount of dependencies. This numbered list below is one example to get the job done fast and smooth, but you may need to do things differently:

1. download and install miniconda https://docs.conda.io/en/latest/miniconda.html

2. create a conda environment with this command: *conda env create -f environment.yml* the environment.yml file can be found in the root directory and the newly created environment will be called HPC_Drug . As the dependencies of any package can change at any time the environment.yml file might not be up to date, in case install the missing packages manually. Of course if you prefer to have more control over the process you can install everything manually (but it would take some time)

3. activate the environment: *conda activate HPC_Drug*

4. install plumed:[2] if you don't need some of the advanced functionalities of plumed (we won't need them) you already installed it while creating the conda environment (good job!), otherwise you can find all the needed information on the plumed website https://www.plumed.org

5. install Orac[3] (they are distributed together): download Orac from it's website http://www.chim.unifi.it/orac and follow the installation guide on the documentation. (As it is a quite challenging task below you will find a little help paragraph for this step)

6. install Gromacs:[4] if you want to use Gromacs instead of Orac as MD program you can install it by following the instructions on the Gromacs website http://www.gromacs.org, in case you have some problems in the compilation process or you need to patch it with plumed (necessary if you want to use a Replica Exchange Method (REM) in older versions, optional in newer versions) I found this blog article very useful https://sajeewasp.com/gromacs-plumed-gpu-linux/ (it is for an old version of Gromacs but still useful).

Installing Orac

- Prerequisites:
  - GNU Make
  - GCC 4.3 or higher
  - (possibly) other Fortran 90 compilers
- Prerequisites for building the parallel version:
  - MPI (Message Passing Interface) libraries and implementation/environment:
    MPICH2 [http://www.mcs.anl.gov/research/projects/mpich2/]
    -or-
    OPENMPI [http://www.open-mpi.org/]
  - also see README_PARALLEL in tests/*/

To build ORAC executable program:

1. change directory to src/
2. type ./configure [options]
3. make

This will create an executable orac in the target directory as specified in [options]. For example, to generate a target for Intel/ifort with OpenMP and MPI support and fftw libraries type:

./configure -Intel -FFTW -OMP -MPI

make

A target "orac" is created in the directory /INTEL-FFTW-OMP-MPI.

To list all configure options, type: ./configure -help.

If you want to use the fftw libraries, you must have these installed in your system. For parallel execution, libfftw3, libfftw3_omp, libfftw3_threads are needed. In Debian/Ubuntu systems these libraries are provided in the packages fftw3 and fftw3-dev. An alternative path for fftw libraries can be specified in the -FFTW configure option [-FFTW[=alt_path]]. The file src/config.H sets the dimensions for the program. The current settings work for the attached tests; the user may have to change this setting for running her/his project.

The program has been tested on Linux and AIX platforms. On other platforms, the configure and Makefile.in files may need some hacking. The package is known to compile and run with gcc 4.8 and Intel Fortran Compiler (10.1 to 14) both in 32- and 64- bit architecture; XL Fortran for AIX (V12.1-V14.1) and XL C/C++ for AIX (V10.1-V12.0); PGI Fortran 2003.

To install the code (locally and for the session):

- change directory to etc/
- type source ORACRC

the command, if successful, prints a list of the available orac commands.

N.B.: compile both the mpi and the non-mpi version of the code (see above) to have orac and orac_mpi in the list.

To run the program tests:

- To run the examples, cd to tests/ and read the README

- The non-mpi program is run from the command line as:

  orac < input-file > output-file

- The mpi program is run from the command line as:

  mpirun -n 9 orac_mpi < input-file > output-file

where orac and orac_mpi are soft-links to the executable in src and "input-file" is provided by the user; the input files in the "test" sub-directory may be used as templates.

ORAC needs additional input files: for example, the files for the Force Field parameters and the molecule topology; more files may be needed, depending on the problem. The files' path must be specified in the main input file. See the manual section "Input to ORAC" for more info.

References

(1) Trott, O.; Olson, A. J. AutoDock Vina: Improving the Speed and Accuracy of Docking with a New Scoring Function, Efficient Optimization, and Multithreading. *J. Comput. Chem.* **2010**, *31*, 455–461.

(2) Tribello, G. A.; Bonomi, M.; Branduardi, D.; Camilloni, C.; Bussi, G. PLUMED 2: New Feathers for an Old Bird. *Comp. Phys. Commun.* **2014**, *185*, 604 – 613.

(3) Procacci, P. Hybrid MPI/OpenMP Implementation of the ORAC Molecular Dynamics Program for Generalized Ensemble and Fast Switching Alchemical Simulations. *J. Chem. Inf. Model.* **2016**, *56*, 1117–1121.

(4) Hess, B.; Kutzner, C.; van der Spoel, D.; Lindahl, E. GROMACS 4: Algorithms for Highly Efficient, Load-Balanced, and Scalable Molecular Simulation. *J. Chem. Theory Comput.* **2008**, *4*, 435–447.
